# Editorial: Advancements and future challenges in molecular diagnostics and therapeutics

**DOI:** 10.3389/fmolb.2025.1620909

**Published:** 2025-05-15

**Authors:** Matteo Becatti, Masoud Mozafari, Roberta Risoluti

**Affiliations:** ^1^ Department of Experimental and Clinical Biomedical Sciences “Mario Serio”, University of Firenze, Firenze, Italy; ^2^ Research Unit of Health Sciences and Technology, Faculty of Medicine, University of Oulu, Oulu, Finland; ^3^ Department of Chemistry, Sapienza University of Rome, Rome, Italy

**Keywords:** diseases, molecular medicine, molecular diagnostics, therapeutics, clinical decision

The field of molecular diagnostics and therapeutics stands at a pivotal juncture. What began as parallel efforts, decoding disease mechanisms on one side and developing targeted treatments on the other, has rapidly matured into an era of convergence. Today, diagnostics are no longer just endpoints in the clinical decision chain; they are embedded in the very fabric of therapeutic design, and *vice versa*. The six studies presented in this Research Topic illustrate this paradigm shift, tracing a common narrative that cuts across disease categories, methodological innovations, and translational aspirations.

At the core of this transformation is the power of molecular systems analysis. Through increasingly sophisticated bioinformatics pipelines and high-throughput omics technologies, researchers are now able to decode the molecular crosstalk underlying complex, multifactorial diseases. In a compelling demonstration of this approach, Chenxuyu et al. explored the shared molecular landscape of asthma and ulcerative colitis—two seemingly distinct inflammatory conditions. By integrating gene expression datasets and applying machine learning models, they identified four hub genes (NOS2, TCN1, CHI3L1, and TIMP1) central to both pathologies. Notably, they proposed repositioning known therapeutic agents such as beclomethasone and PD 98059, leveraging drug-target docking simulations. This study exemplifies how transcriptomic cross-disease mining can unearth novel diagnostic biomarkers while simultaneously informing therapeutic hypotheses.

The harnessing of genomic information for real-time surveillance is equally emblematic of this new integrated frontier. Mazzarella et al. conducted a regional genomic analysis of SARS-CoV-2 Omicron variants in Southern Italy, covering the critical phase between December 2021 and February 2023. Using next-generation sequencing, they mapped 15 distinct Omicron subvariants and correlated their molecular profiles—particularly mutations in the spike protein—with symptomatology and geographic diffusion. Their findings illustrate how viral genomics not only enhances our understanding of pathogen evolution but serves as a crucial diagnostic and predictive framework for public health responses, vaccine adaptation, and antiviral strategy.

Moving from diagnosis to treatment, Baggio et al. explored the therapeutic potential of polydatin, a natural polyphenol, in calcium pyrophosphate deposition disease, a common cause of pseudogout. Through a combination of *in vitro* and *in vivo* models, they demonstrated that polydatin significantly attenuates inflammatory responses by modulating the SIRT1 pathway and inhibiting CCR1 signaling. Importantly, the compound affected multiple inflammatory cytokines, chemokines, and angiogenic factors, revealing a multi-targeted mechanism of action. Their work underscores the emerging trend of leveraging natural compounds as multi-modal therapeutic agents, guided by molecular pathway insights rather than trial-and-error pharmacology.

On the diagnostic innovation front, Adamiec-Mroczek et al. made a notable contribution by designing a custom ELISA assay to measure neutrophil elastase, a protease implicated in tissue damage during both acute COVID-19 and post-COVID syndrome. Their assay was validated in patients with diabetic nephropathy, where elevated neutrophil elastase levels were predictive of advanced post-COVID syndrome manifestations. By ensuring both technical precision and economic feasibility, this study demonstrates the value of clinically scalable diagnostics rooted in molecular markers, especially for chronic sequelae that are otherwise difficult to monitor using conventional tools.

Segura-Ulate et al. approached diagnostics from a different angle, addressing the global need for cost-effective, accessible, and rapid testing platforms for COVID-19. By comparing RT-LAMP and RT-qPCR methodologies using both nasopharyngeal swabs and saliva samples, they evaluated how changes in sample Research Topic and RNA extraction protocols influenced sensitivity and specificity. Their results confirm that simplified testing workflows, particularly those based on LAMP paired with traditional RNA extraction from saliva, can offer a viable alternative to standard PCR, especially in resource-constrained or high-throughput environments. Yet they also caution against over-simplification, highlighting the trade-offs in diagnostic accuracy when protocols are stripped down too far.

In a final demonstration of diagnostics-therapeutics integration, Chu et al. investigated the role of miRNA-mRNA networks in cerebral vasospasm following subarachnoid hemorrhage. Their bioinformatics-driven identification of differentially expressed miRNAs (Let-7a-5p, miR-24–3p, miR-29a-3p, and miR-132–3p) and their mRNA targets (CDK6 and SLC2A1) were experimentally validated in both patient tissue samples and cultured smooth muscle cells. Their work provides mechanistic insights into the development of cerebral vasospasm and points toward potential molecular targets for early intervention. More broadly, it exemplifies how multi-layered transcriptomic analysis can translate into clinically relevant strategies for managing acute neurological conditions.

Across all six contributions, a unifying theme emerges: a bidirectional model of translation, where diagnostics inform treatment and therapeutic outcomes refine diagnostic development. The studies reflect a field increasingly reliant on multi-omics integration, machine learning, and network-based approaches to understand disease not as a static entity, but as a dynamic system of interacting molecular events. But even as the science advances, so too do the challenges. High-throughput data alone cannot guarantee clinical impact. Issues of data standardization, reproducibility, and real-world validation remain pressing. Moreover, the equitable implementation of these innovations, especially in under-resourced health systems, requires careful consideration of economic models, infrastructure, and education. The integration of artificial intelligence, while promising, also raises ethical and regulatory concerns, including transparency in decision-making and biases in data training sets.

This Research Topic reflects not a destination, but a trajectory, a movement toward molecular integration as a foundation for future medicine. By linking benchside discoveries with bedside applications, the authors in this Research Topic offer a roadmap for turning complexity into clarity, and insight into impact.

